# Intrinsic neural network dysfunction in quiescent Crohn’s Disease

**DOI:** 10.1038/s41598-017-11792-y

**Published:** 2017-09-14

**Authors:** Anne K. Thomann, Martin Griebe, Philipp A. Thomann, Dusan Hirjak, Matthias P. Ebert, Kristina Szabo, Wolfgang Reindl, Robert C. Wolf

**Affiliations:** 1 Department of Medicine II, Medical Faculty Mannheim, Heidelberg University, 68167 Mannheim, Germany; 2 Department of Neurology, Medical Faculty Mannheim, Heidelberg University, 68167 Mannheim, Germany; 3 Department of General Psychiatry, Heidelberg University Hospital, 69115 Heidelberg, Germany; 4 Department of Psychiatry and Psychotherapy, Central Institute of Mental Health, Medical Faculty Mannheim, Heidelberg University, 68159 Mannheim, Germany; 5 Center for Mental Health, Odenwald District Healthcare Center, 64711 Erbach, Germany

## Abstract

Psychological factors and comorbidities play an important role in inflammatory bowel diseases. Such comorbidity could be associated with a specific neural phenotype. Brain regions associated with emotion regulation and self-referential processing, including areas assigned to the “default mode network” (DMN), could be promising candidates in this regard. We investigated the functional integrity of multiple intrinsic neural networks in remitted patients with Crohn’s disease (CD) and sought to establish relationships between neural network connectivity and psychiatric symptoms. Fifteen CD patients in remission and 14 controls were investigated. We employed resting-state functional magnetic resonance imaging (fMRI) at 3 Tesla followed by a spatial Independent Component Analysis for fMRI data. Abnormal connectivity in CD patients was observed in DMN subsystems only (p < 0.05, cluster-corrected). Increased connectivity was found in the anterior cingulate and left superior medial frontal gyrus (aDMN) and the middle cingulate cortex (pDMN). Middle cingulate activity showed a significant association with anxiety scores in patients (p = 0.029). This study provides first evidence of selectively disrupted intrinsic neural network connectivity in CD and suggests abnormalities of self-referential neural networks. An increased sensitivity to self-related affective and somatic states in CD patients could account for these findings and explain a higher risk for anxiety symptoms.

## Introduction

Inflammatory bowel diseases (IBD) were considered classical “psychosomatic” disorders until the second half of the 20th century, when the concept of an autoimmune background emerged. Today, IBD are assumed to result from an inappropriate activation of intestinal mucosal immunity in genetically susceptible hosts. Yet the immunological process alone is insufficient to explain the phenotypic diversity of IBD in terms of its clinical expression, where complex interactions of psychological, biological and social factors have to be taken into account. For instance, longitudinal studies yield increasing evidence that stress and mood disorders can adversely affect the course of IBD^1^ by unclear pathomechanisms. Rodent models of IBD have shown a direct influence of psychological stress on bowel inflammation. A higher incidence of anxiety^2, 3^, depression^3, 4^ and cognitive dysfunction^5, 6^ has been described in patients with IBD. Psychotherapy has been shown to influence quality of life but not disease activity in individuals with IBD^7^.

Although acknowledged, the significance of brain-gut-interactions in IBD is poorly elucidated at present^8^. Possible alterations of brain morphology have increasingly attracted scientific interest over the past years. Studies using magnetic resonance imaging (MRI) have reported structural changes^9, 10^ that have also been linked to psychological factors^11^. More recently, functional MRI (fMRI) has been employed to investigate neural activity in IBD^12–14^. The extant functional data suggest regionally confined activation differences in areas associated with regulation of distress and affect, as well as in cortical regions subserving self-referential processing. In patients with irritable bowel syndrome (IBS), some of these changes in brain activity have been also linked to the expression of psychiatric symptoms, e.g. anxiety and depression^15^, suggesting a neural phenotype that may indicate psychiatric vulnerability. Such neural markers may be detected by task-based functional neuroimaging, as pursued by the vast majority of studies in patients with IBD so far. An alternative technique for the *in-vivo* measurement of neural coupling is the assessment of intrinsic brain activity using “resting-state fMRI” (rs-fMRI), an approach that offers a rich source of information on intrinsic spatiotemporal brain connectivity dynamics. The concept of intrinsic neural networks in the brain is a relatively young topic in systems neuroscience. Intrinsic networks are thought to reflect fundamental functional characteristics of the brain in the absence of explicit external stimulation^16, 17^. Such networks are characterized by neuroanatomically distinct spatial patterns exhibiting synchronous spontaneous blood-oxygen-level dependent (BOLD) signal fluctuations “at rest”, in contrast to states where processing of external stimuli is required. Multiple spatiotemporally distinct neural systems have been robustly identified across multiple independent data sets; these include spatially stable patterns of sensorimotor, auditory, visual and several discrete lateral and medial-prefrontal systems^18, 19^. Importantly, such resting-state networks (RSN) show a close spatial correspondence to activity patterns underlying a wide range of sensory, affective and higher cognitive functions^18, 20^.

A specific RSN which has been consistently related to affective and cognitive self-referential processing is the so-called default mode network (DMN), a set of brain regions with an inter- and intraindividually stable pattern that show higher activity at rest and lower activity during explicit cognitive demand. The anatomical structures of the DMN essentially include cortical midline regions such as the anterior and posterior cingulate cortices, the anterior medial prefrontal cortex, the precuneus, and bilateral inferior parietal regions^17^. Altered functional connectivity of regions attributed to the DMN has been shown to characterize various mental disorders including depression and anxiety disorders^21, 22^. A role of the DMN as a potential biomarker for evaluating disease progress or therapeutic measures has recently drawn attention of likewise clinicians and neuroscientists^23–25^. For instance, in patients with major depression, DMN abnormalities were shown to correlate with symptom severity^26^ and to change as a function of antidepressive treatment^27^.

In this study, we investigated the functional integrity of multiple neural networks in remitted patients with Crohn’s disease (CD). We also sought to establish relationships between neural network connectivity and psychiatric symptoms, such as anxiety and depression. We used rs-fMRI and multivariate statistical techniques for fMRI data analysis (i.e. Independent Component Analysis, ICA) to investigate RSN connectivity differences between remitted CD patients and healthy controls. Driven by the extant evidence suggesting regionally confined brain activity changes in individuals with IBD, we specifically focused on distinct networks associated with self-referential processing and self-regulation (i.e. DMN) on the one hand and frontoparietal systems which have been previously associated with higher order cognition (such as executive function or working memory) and comprise areas along the intraparietal sulcus and dorsal premotor cortex on the other hand^18, 20^. With respect to DMN integrity, we focused on two distinct subsystems following an anterior/posterior-dissociation, since recent research supports the idea that the DMN is not a unitary system, but rather is composed of smaller and distinct functional subsystems that interact with each other. DMN subsystems include an anterior (predominantly medial prefrontal and anterior cingulate) and a posterior (predominantly precuneus and posterior cingulate) subsystem^28^. The interplay among subsystems within the DMN is thought to be crucial for the physiological function of this system^29^. In line with the extant data suggesting an involvement of DMN function in IBS, and given the chronicity of IBD with respect to recurrent pain burden, the higher incidence of psychological factors and the therapeutic significance of stress reduction in a majority of patients with IBD as well as symptom similarities between IBS and IBD (i.e. abdominal pain, cramps, diarrhea, bloating), we predicted that CD patients in full remission would show altered DMN function in anterior cingulate and medial prefrontal regions compared to healthy controls. Given that cognitive dysfunction has been documented in individuals with IBD/CD and seems to be influenced by factors like depression or rumination in these patients^5, 6, 30^, we also expected that frontoparietal networks, i.e. systems previously related to goal-directed behavior, attention and executive function^20^, would show abnormal lateral prefrontal connectivity in CD patients compared to healthy controls. In addition, using a ‘functional network connectivity’ approach^31^ we investigated between-network coupling, i.e. the functional interplay between DMN subsystems and frontoparietal networks, and potential between-network strength differences between patients and controls. Finally, we sought to investigate potential associations between brain activity and clinically relevant measures such as anxiety, depression, fatigue and cognitive function in order to determine a possible neuronal phenotype of CD patients with specific disease-related psychological symptoms.

## Results

### Participants

We observed no significant differences between patients and controls in terms of age, gender, education, cardiovascular risk factors, neurological examination results and psychometric values, with trends towards higher depression and fatigue scores in patients, (Table 1). One patient and 1 HC had anxiety-subscores above the cutoff-value, indicating clinically relevant anxiety.

**Table 1:** Demographics and clinical characteristics of CD patients and healthy controls (HC).

	CD (n = 15)	HC (n = 14)	*P* value
Gender; male/female	6/9	6/8	1
Age, years; mean (SD)	41.3 (3.5)	42 (13)	0.842^*^
Education, years; mean (SD)	14.7 (0.7)	15 (2.7)	0.681^*^
BMI; mean (SD)	27.1 (4.9)	26.3 (3)	0.8^*^
Cardiovascular risk factors; n	9	8	1^†^
smokers; n	7	7	1
packyears; mean (SD)	13.6 (9)	15.3 (12.5)	0.774^*^
hypertension; n	4	3	1^†^
BDI; mean (SD)	7.5 (4.8)	4.9 (5)	0.107^‡^
HADS-A; mean (SD)	5.9 (3.9)	4.6 (2.7)	0.407^‡^
WEIMuS; mean (SD)	16.7 (12.5)	11.9 (15.7)	0.162^‡^
MoCA; mean (SD)	28.1 (1.4)	27.9 (2.3)	1^‡^
CDAI; mean (SD)	33 (38)	—	—
Disease duration, years; mean (SD)	18.1 (13.9)	—	—
Fecal Calprotectin, g/kg; mean (SD)	40.5 (42.2)	—	—

BMI, Body mass index; BDI, Beck Depression Inventory; CD, Crohn’s Disease; CDAI, CD Activity Index; HADS-A: Hospital Anxiety and Depression Scale, anxiety subscore; HC, healthy controls; MoCA, Montreal Cognitive Assessment; SD, standard deviation; WEIMuS, Würzburger Erschöpfungsinventar bei Multipler Sklerose (Wuerzburger Fatigue Inventory for Multiple Sclerosis).

^*^Student’s t-test; ^‡^Mann-Whitney-U-test, ^†^Fisher’s exact test, two-tailed.

### MRI results

**Figure 1:**
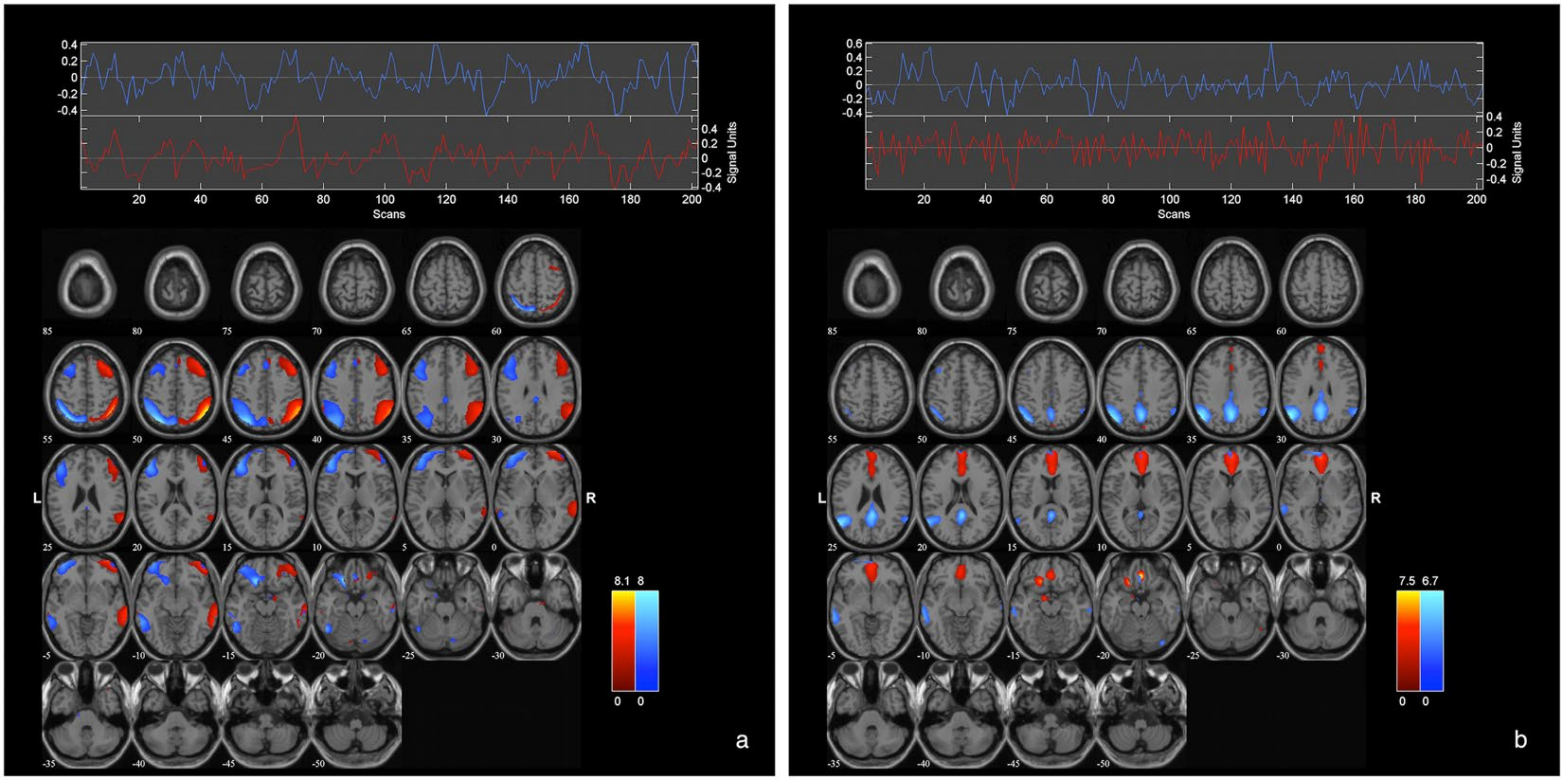
Patterns of functional connectivity within frontoparietal (**a**) and DMN (**b**) components. Common to controls and patients, the figure displays independent components (IC) and their corresponding time courses, as identified by the group ICA. The composite IC maps were obtained using the graphical output function provided by the GIFT software (http://mialab.mrn.org/software/gift/index.html). The color bars indicate Z-values. For illustrative purposes, IC’s are thresholded at Z = 2.5.

From a total of 27 estimated independent components (ICs), four RSN of interest were identified using spatial sorting (see section 2.3.2). These networks included left and right lateral frontoparietal cortical (FPC) regions, as well as anterior and posterior cortical midline regions, as ascribed to anterior and posterior DMN subsystems, respectively (see also Fig. 1 and Tables 2 and 3 for detailed anatomical denominations, stereotaxic coordinates, Z-scores and cluster volumes).

**Table 2:** Anatomical denominations, stereotaxic coordinates, Z-scores and cluster volumes for the anterior and posterior DMN.

*Brain region*	*Brodmann Area*	*L*	*R*	
		*Z-score/coordinates (x,y,z)*	*Z-score/coordinates (x,y,z)*	*volume (cc) L/R*
**anterior DMN**				
Inferior Frontal Gyrus	11, 47	6.9 (−20, 22, −16)		0.9/0.0
Rectal Gyrus	11	6.5 (−4, 34, −19)	4.9 (4, 36, −19)	0.1/0.1
Middle Frontal Gyrus	11	6.2 (−20, 26, −16)		0.3/0.0
Medial Frontal Gyrus	9, 10, 11, 25	5.7 (−4, 30, −18)	5.4 (4, 32, −18)	4.0/3.3
Orbital Gyrus	11	5.0 (−4, 38, −19)	3.2 (4, 40, −19)	0.1/0.1
Anterior Cingulate	10, 24, 32	4.9 (−4, 48, −2)	4.7 (4, 49, −1)	2.8/2.8
Parahippocampal Gyrus		4.1 (−14, −5, −13)		0.1/0.0
Superior Frontal Gyrus	9, 10	3.1 (−4, 56, 25)	3.1 (4, 56, 25)	0.1/0.1
Precuneus	7		3.1 (6, −77, 44)	0.0/0.1
Fusiform Gyrus			3.1 (51, −59, −17)	0.0/0.1
**posterior DMN**				
Supramarginal Gyrus	40	6.7 (−53, −61, 33)	4.0 (59, −55, 30)	3.0/0.7
Angular Gyrus	39, 40	6.5 (−51, −62, 36)	3.5 (57, −57, 34)	1.5/0.1
Superior Temporal Gyrus	22, 39	6.4 (−55, −59, 29)	3.4 (59, −55, 27)	1.7/0.1
Inferior Parietal Lobule	7, 39, 40	6.0 (−50, −62, 40)	3.4 (59, −53, 38)	3.4/0.2
Precuneus	7, 19, 31, 39	5.9 (0, −58, 36)	5.1 (2, −51, 34)	4.2/2.1
Middle Temporal Gyrus	21, 39	5.9 (−53, −63, 29)	3.8 (65, −26, −14)	2.6/0.1
Posterior Cingulate	23, 29, 30, 31	5.5 (0, −45, 23)	4.8 (4, −45, 24)	1.4/0.7
Cingulate Gyrus	31	5.4 (0, −45, 26)	4.8 (2, −45, 30)	2.0/1.0
Superior Parietal Lobule	7	4.5 (−36, −68, 46)		0.4/0.0
Cuneus	7	4.3 (−2, −66, 33)	3.9 (4, −64, 33)	0.1/0.1
Medial Frontal Gyrus	10, 25	4.2 (−10, 60, −5)		0.4/0.0
Superior Frontal Gyrus	10	3.3 (−12, 62, −1)		0.3/0.0

For the networks shown in Fig. 1 (right), voxels >Z = 3.0 were converted from MNI to Talairach coordinates and coupled with the Talairach Daemon database to provide anatomical labels. Maximum Z-values and stereotaxic coordinates (x, y, z) are provided for each hemisphere (left = L, right = R). The volume of voxels in each area is provided in cubic centimeters (cc).

**Table 3:** Anatomical denominations, stereotaxic coordinates, Z-scores and cluster volumes for the left and right frontoparietal networks.

*Brain region*	*Brodmann Area*	*L*	*R*	
		*Z-score/coordinates (x,y,z)*	*Z-score/coordinates (x,y,z)*	*volume (cc) L/R*
**left frontoparietal network**				
Superior Parietal Lobule	7	8.0 (−32, −67, 49)		3.6/0.0
Precuneus	7, 19	7.7 (−28, −69, 50)		3.3/0.0
Middle Frontal Gyrus	8, 9, 10, 11, 46	7.5 (−20, 24, −16)		9.2/0.0
Inferior Parietal Lobule	7, 39, 40	6.7 (−40, −64, 47)		6.1/0.0
Inferior Frontal Gyrus	10, 11, 45, 46, 47	6.1 (−20, 21, −16)		4.1/0.0
Superior Frontal Gyrus	8, 10, 11	4.9 (−36, 58, −1)		1.5/0.0
Middle Temporal Gyrus	21, 37	4.9 (−59, −49, −9)	3.6 (65, −26, −14)	1.3/0.1
Fusiform Gyrus	37	4.8 (−53, −57, −16)		0.4/0.0
Inferior Temporal Gyrus	20, 21, 37	4.7 (−55, −55, −12)		0.7/0.0
Precentral Gyrus	9	3.7 (−46, 23, 34		0.2/0.0
Postcentral Gyrus	40	3.6 (−55, −36, 48)		0.2/0.0
Uncus	34	3.6 (−18, 1, −22)		0.1/0.0
Medial Frontal Gyrus	11, 25	3.5 (−12, 28, −15)		0.3/0.0
Angular Gyrus	39	3.3 (−34, −60, 36)		0.3/0.0
**right frontoparietal network**				
Superior Parietal Lobule	7, 40		8.3 (42, −60, 51)	0.0/2.0
Inferior Parietal Lobule	7, 39, 40	3.1 (−55, −46, 47)	8.1 (44, −54, 52)	0.1/8.4
Superior Frontal Gyrus	8, 9, 10, 11		6.0 (32, 62, −5)	0.0/3.5
Middle Frontal Gyrus	6, 8, 9, 10, 11, 46, 47		5.7 (38, 60, −8)	0.0/10.0
Supramarginal Gyrus	40		5.4 (59, −53, 36)	0.0/2.8
Postcentral Gyrus	2, 40		5.3 (53, −34, 51)	0.0/0.4
Middle Temporal Gyrus	21		5.2 (67, −37, −3)	0.0/3.4
Precuneus	7, 19		5.1 (40, −68, 42)	0.0/0.6
Angular Gyrus	39		4.6 (53, −56, 36)	0.0/0.7
Inferior Frontal Gyrus	47		4.2 (50, 44, −11)	0.0/0.2
Superior Temporal Gyrus	21, 39		4.2 (59, −55, 29)	0.0/0.3
Precentral Gyrus	9		3.9 (46, 21, 36)	0.0/0.4
Medial Frontal Gyrus	25	3.1 (−2, 28, −18		0.1/0.0

For the networks shown in Fig. 1 (left), voxels > Z = 3.0 were converted from MNI to Talairach coordinates and coupled with the Talairach Daemon database to provide anatomical labels. Maximum Z-values and stereotaxic coordinates (x, y, z) are provided for each hemisphere (left = L, right = R). The volume of voxels in each area is provided in cubic centimeters (cc).

**Figure 2:**
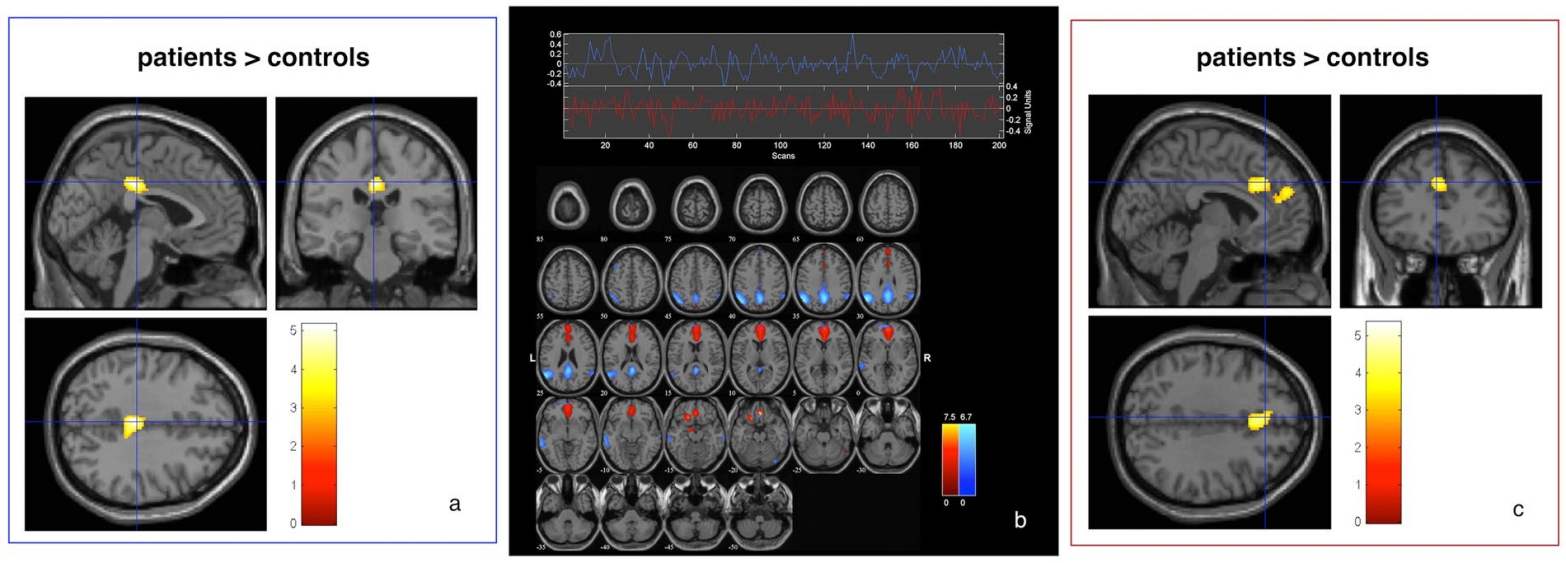
Functional connectivity differences between patients and controls within spatially distinct DMN subsystems. Connectivity differences between patients and controls within the posterior (**a**) and anterior (**c**) DMN. Results are derived from 2^nd^ level two-sample t-test models covaried for age, gender and mean framewise displacement, p < 0.005 (uncorrected at the voxel level), p < 0.05 corrected for spatial extent. The color bars indicate Z-values. (**b**): spatial patterns of the DMN subsystems identified by spatial sporting. Blue: posterior DMN. Red: anterior DMN. Patterns of functional connectivity within frontoparietal (left) and DMN (right) components. Common to controls and patients, the figure displays independent components (IC) and their corresponding time courses, as identified by the group ICA. Composite IC maps were obtained using the graphical output function provided by the GIFT software (http://mialab.mrn.org/software/gift/index.html). The color bars indicate Z-values. For illustrative purposes, IC’s are thresholded at Z = 2.5.

Within the posterior DMN, patients exhibited increased functional connectivity of the middle cingulate cortex [MCC] (x = −2, y = −26, z = 36, Z = 4.19, k = 328 voxels). Within the anterior DMN, patients exhibited increased connectivity in a cluster comprising the anterior cingulate (ACC) and left superior medial frontal gyrus (x = 0, y = 24, z = 30, Z = 4.30 and x = −4, y = 52, z = 26, Z = 3.38 k = 578 voxels); see also Fig. 2.

Differences between patients and controls were not detected for both frontoparietal systems.

### Functional network connectivity

Functional network connectivity analyses revealed significant between RSN coupling between the frontoparietal and DMN systems RSN in controls and patients (Fig. 1, supplementary data). The functional coupling, i.e. the significant maximal correlations and the lag-values, between patients and controls, did not significantly differ.

### Relationships between functional connectivity and clinical measures

Anxiety scores from the Hospital Anxiety and Depression Scale (HADS, German version) and MCC connectivity showed a significant positive correlation within the CD group (r = 0,562, p = 0,029), but not in HC (r = 0,226, p = 0,437).

No significant correlations were observed between ACC or MCC connectivity for any of the other clinical variables including depression (Beck Depression Inventory II, BDI-II), fatigue („Würzburger Erschöpfungsinventar bei Multipler Sklerose/Wurzburger Fatigue Inventory for MS, WEIMuS), disease duration, or disease activity (Crohn’s Disease Activity Index, CDAI).

## Discussion

In this study, using a resting-state fMRI approach we investigated the functional integrity of multiple intrinsic neural networks in remitted patients with CD. We sought to establish relationships between neural network connectivity and psychiatric symptoms, such as anxiety and depression. Three main findings emerged: First, CD patients exhibited abnormal DMN connectivity compared to controls, whereas frontoparietal networks did not show significant differences between the groups. Second, between-network coupling did not differ between the groups. Third, a relationship between anxiety levels and MCC connectivity was observed in CD patients, but not in controls.

We provide evidence of intrinsic neural network dysfunction as reflected by connectivity changes in the aDMN and pDMN, specifically in regions of the ACC and MCC. This is in line with previous studies of brain function in IBD, which have predominantly shown limbic dysfunction that could predispose patients to comorbid mental disorders or sub-clinical psychiatric symptoms including anxiety and depression. For instance, recent task-based studies reported an inadequate habituation to stress^32^, a decreased sensitivity to positive emotions^13^ and exaggerated brain responses in anticipation to visceral discomfort compared to healthy individuals^12^. These neurobiological observations show deficits of affect and stress regulation in IBD, at least in the presence of extrinsic stimulus processing. The cingulate cortex is a limbic brain region that has been examined in numerous structural and functional studies. Its involvement in fear^33^, visceral pain processing^12, 34^ depression^35^ and self-monitoring processes is widely accepted^36, 37^.

It is important to note that DMN systems were selectively impaired in this CD sample in contrast to networks predominantly involved in external goal-directed processes, i.e. the lateral frontoparietal systems investigated in this study. The DMN is known to play an important role in mnemonic and selfreferential processes^17, 38^, as much as it has been associated with affective regulation and control^39^. Patients with chronic diseases live with the constant knowledge that disease activity can (re-)occur at anytime, which can lead to increased selfmonitoring. The aDMN seems to be involved in (repetitive) selfreflective actions such as rumination^26^ and the ACC plays an important role for the processing of visceral stimuli^40^. The observed regionally increased coupling in the aDMN could represent an increased tendency to monitor visceral symptoms compared to HC. Recent evidence suggests DMN alterations in IBS and chronic pain conditions such as fibromyalgia, chronic back pain and ankylosing spondylitis^41–43^. Recently, decreased functional connectivity within distinct regions of the DMN has been shown in patients with IBS, where brain activity was associated with overall symptom severity and anxiety levels^15, 43^. Interestingly, in a task-based fMRI study that applied pain stimuli in the form of rectal distensions to a group of IBS patients and healthy controls, anxiety scores correlated significantly with higher pain induced activation of the anterior MCC and the perigenual ACC, regions that have been described as DMN key hubs^44^.

Considering putative functions of the DMN, it is not surprising to find alterations in chronic somatic disorders, but it is unclear whether these changes represent specific disease signatures or a common sign of chronic illness. For instance, studies on patients with chronic pain showed decreased brain activity in the medial prefrontal cortex^45^, a higher functional connectivity between the DMN and the insular cortex^42, 45, 46^ and between the DMN and the salience network^47^. A recent study on fibromyalgia showed an alteration of DMN connectivity that involved the aMCC^48^, patients with chronic tinnitus show decreased connectivity within the pDMN, in an area corresponding to the precuneus^49^. While it is possible that DMN dysfunction may be detected across a variety of chronic somatic disorders, regional deficits may vary as a function of symptom expression (e.g. pain vs. auditory discomfort), and disorder-specific between-network interactions may be detectable as well (e.g. interactions between the DMN and the networks processing salience).

The intact frontoparietal network function, as well as the absence of group differences in between-network coupling in this patient sample is in line with the observed normal cognitive function. In this sample, DMN dysfunction had no impact on between-network strength, as it had been previously suggested e.g. in mental disorders with prominent cognitive deficits, such as attention-deficit/hyperactivity disorder^50^. Since we investigated remitted CD patients presenting with intact cognitive function (as suggested by MoCA (Montral Cognitive Assessment) -scores), it remains open at this stage of research whether DMN and frontoparietal network integrity and coupling is selectively altered in patients with different stages of active disease. For instance, in patients with higher disease activity levels who may well show cognitive deficits^6^, disturbed DMN/frontoparietal network coupling could be a likely neural mechanism which may explain the occurrence of cognitive symptoms. Yet although such a mechanism may appear plausible, specific hypotheses remain to be addressed by future research.

Relationships between neural integrity and psychiatric symptoms have been previously reported in patients with IBD. For instance, Bao and colleagues^11^ observed structural brain changes in terms of lower cortical thickness in brain regions including the cingulate cortex that were significantly less prominent after correction for anxiety and depression scores. More recently, intrinsic brain dysfunction has been shown in individuals with irritable bowel syndrome, where effects of anxiety and depression were observed in anterior and dorsal regions of the cingulate cortex^15^. The current findings confirm these observations and suggest a relationship between anxiety levels and increased cingulate cortical connectivity in CD patients. Although a higher prevalence of depression, anxiety and cognitive disturbances has been described in IBD patients^2, 4, 5, 12^, the samples investigated in this study did not differ significantly in any clinical variable including anxiety and cognition scores. This could partly be explained by the fact that all our patients were in stable remission and had a relatively mild disease course (i.e. had not been treated with biologics before), as some psychological comorbidities in IBD are known to be driven by disease activity^3, 4, 51^. Nevertheless, despite similar levels of anxiety and depression, CD patients in this study exhibited regionally abnormal connectivity in both DMN subsystems. Moreover, within the pDMN the MCC was correlated with anxiety in the patient group, but such relationship was not found in healthy controls. This suggests that the observed abnormality of the pDMN may be linked to anxiety levels in CD patients, yet in the absence of significantly heightened anxiety levels in patients compared to healthy individuals, anxiety alone is insufficient to explain these findings. Eventually, at the neural level, it is unclear whether patients with IBD and anxiety or depression present a specific neural phenotype that can be distinguished from other persons with these symptoms, i.e. individuals suffering from anxiety disorders. In these persons, DMN abnormalities have been frequently reported^52^, so that transnosologic neural mechanisms appear possible. In the absence of direct comparisons, i.e. contrasting individuals with specific anxiety disorders to CD patients, robust conclusions cannot be made at this stage.

We acknowledge limitations to this study, such as the relatively modest sample size and the lack of a clinical comparison sample e.g. including CD patients with active disease. Since we included remitted patients only, further conclusions considering patients presenting with various degrees of disease activity cannot be drawn. Also, it remains open to which extent findings derived from a resting-state condition may also apply to task-based data. HADS and BDI have their own limitations when used as screening tools for anxiety and depression. Furthermore, the MoCA only briefly taps into various cognitive functions, so that a comprehensive neuropsychological evaluation may yield additional benefits. We also infer from cross-sectional data, so that further research is warranted to determine potential changes of brain connectivity over time as a function of disease activity or psychiatric symptom load. Eventually, inference is made from resting-state data. Thus, at this stage of research it remains unclear how external demand could modulate DMN function and between-network coupling in CD patients. Further research is needed to determine possible interactions between abnormal resting-state activity and processes driven by extrinsic demand, such as emotion regulation in the presence of stress or goal-directed cognition.

## Conclusions

This study provides first evidence of abnormal intrinsic neural network connectivity in patients with CD in remission. The data suggest a prominent role of selfreferential networks (i.e. DMN) in contrast to systems predominantly involved in goal-directed behaviour (i.e. frontoparietal networks). The relationship between cingulate cortical activity and anxiety levels in patients also raises the possibility that increased somatosensory self-awareness may be associated with disease-related distress and, at least in vulnerable individuals, also with potentially increased psychiatric symptom burden. Stress, depression and anxiety may influence the course of IBD, not only directly by modulating inflammation, but also indirectly by affecting treatment adherence or the motivation to seek medical help. Increased selfmonitoring and impaired selfregulation could lead to anxiety or depression and thus could be a target for psychotherapy even in the absence of clinically relevant symptoms. The findings of this study further strengthen the notion that abnormal selfmonitoring processes and a potential neural vulnerability to anxiety disorders should be considered in the treatment of IBD patients.

## Methods

The study was approved by the local ethics committee (Medical Faculty of Mannheim, University of Heidelberg, Germany) and carried out in accordance with the ethical standards as described in the Declaration of Helsinki (1975, revised in 2008). All participants gave their written informed consent after a thorough explanation of the study design by the investigator.

### Participants

Fifteen CD patients from the outpatient clinic for IBD patients at the university medical center Mannheim were evaluated. Data regarding disease duration and course, medication, clinical variables including cardiovascular risk factors (i.e. smoking, hypertension, diabetes or hyperlipidemia) and other diseases were collected and patients were asked to complete a patients’ diary for Crohn’s Disease Activity Index (CDAI) evaluation in the 7 days prior to the examination. Inclusion criteria for patients were as follows: age between 18 and 65 years, Crohn’s disease with an endoscopic and/or histological diagnosis confirmation at least 2 years prior to inclusion, disease in remission for at least 6 months, a CDAI <150 and right-handedness. Patient-specific exclusion criteria were: active disease as indicated by a CDAI >150 or use of steroids or biologics in the last 6 months, an elevated CRP (>5 mg/dl) or fecal Calprotectin >150 μg/g. The healthy control group consisted of 14 volunteers matched for age, gender, education, BMI and cardiovascular risk factors. In both groups, individuals with a history of neurological, psychiatric disease or malignancies or with MRI contraindications were excluded from participation. All individuals underwent a neurological examination by an experienced neurologist including cognition-testing and completed 3 validated questionnaires regarding depression (Beck Depression Inventory, BDI-II^53^), anxiety (Hospital Anxiety and Depression Scale, HADS^54^, german version) and fatigue. Fatigue was assessed using the Würzburger Erschöpfungsinventar bei Multipler Sklerose (Wurzburger Fatigue Inventory for MS, WEIMuS)“^55^”. In addition, all individuals underwent a test of global cognitive function, as provided by the Montreal Cognitive Assessment (MoCA)^56^.

### MRI data acquisition

The MRI session was performed on a 3 T MAGNETOM Skyra whole body MR scanner (Siemens Medical Solutions, Erlangen, Germany) using a 20-channel head/neck coil.

T2*-weighted images were obtained using echo-planar imaging in an axial orientation (TR = 2210 ms, TE = 23 ms, FoV = 220 × 220 mm², matrix size = 96 × 96, voxel size = 2.3 × 2.3 × 2.3 mm^3^, flip angle = 90°, bandwidth = 1270 Hz/px, parallel acquisition technique GRAPPA acceleration factor 2, slice thickness = 2.3 mm, gap = 0.7 mm, recorded in descending order). Within a scanning session 210 volumes were acquired. MRI scanning was carried out in darkness. Participants were explicitly instructed to relax without falling asleep, to keep their eyes closed, not to think about anything special and move as little as possible. Adherence to these instructions was verified by verbally contacting participants immediately after the resting-state scan, prior to structural data acquisition. No task-based scanning was included in the protocol.

### MRI data analysis

#### Spatial analysis

Data preprocessing was performed with SPM8 (http://www.fil.ion.ucl.ac.uk/spm) and MATLAB 7.3 (MathWorks, Natick, MA). Prior to data processing, the first 8 volumes of the time-series were discarded. The remaining functional images were corrected for motion artifacts, co-registered to the individual T1 images, spatially normalized using DARTEL and smoothed with a 9 mm FWHM isotropic Gaussian kernel. A spatial ICA was then computed on the entire data set using the “Group ICA for fMRI Toolbox” [GIFT; http://mialab.mrn.org/software/gift] (Correa *et al*. (2005). “Comparison of blind source separation algorithms for FMRI using a new Matlab toolbox: GIFT.” Proc IEEE Int Conf Acoustics, Speech, Signal Processing **5**: 401–404).

To increase the stability of the components, we used the ICASSO algorithm^57^ after repeating the ICA estimation 20 times with bootstrapping and permutation. The dimensionality of the functional data was reduced using Principal Component Analysis alternated with data concatenation across subjects, resulting in one aggregate mixing matrix. An ICA decomposition using the Infomax algorithm was used. The “minimum description length’” criteria^58^ were used to estimate the order selection; 27 Independent Components (ICs) were estimated. These ICs were used for a back reconstruction into individual ICs using the aggregate mixing matrix created during the dimensionality data reduction steps. The individual ICs consisting of individual spatial independent maps and time-courses were spatially sorted using a-priori masks, as defined by the Automatic Anatomical Labeling (AAL) atlas^59^ and as provided by the GIFT toolbox. To identify and select ICs/RSN of interest for further between-group analyses, two masks were used for spatial sorting: 1^st^ a “prefrontal mask” was compted using the AAL, comprising the bilateral superior, inferior, middle and medial frontal gyri. 2^nd^, a spatial default mode network (DMN) mask was used, as provided by GIFT. RSN that were significantly (p < 0.001) spatially correlated with these masks were chosen for 2^nd^ level analyses. The two masks described above were only used to identify networks of interest. They were not used to constrain 2^nd^ level within- and between-group analyses on a certain set of brain regions. Voxelwise one-sample t-tests against the null hypothesis of zero magnitude were used to calculate within-group maps for each of the four RSN identified using the spatial sorting process. Between-group comparisons were performed using two-sample-t-tests covaried for age and gender. Further, using derivatives of the six rigid-body realignment parameters estimated during standard volume realignment individual mean framewise displacement (FD) values were calculated^60^. We computed FD values by summing up absolute motion differences between adjacent volumes, i.e., absolute differences in 3 translations and rotations. FD was included as additional covariate of no interest in all analyses. A threshold of p < 0.005, uncorrected at the voxel level, p < 0.05 corrected for spatial extent^61^, was chosen for all 2^nd^ level between-group comparisons. Stereotaxic coordinates are reported as coordinates of cluster-maxima in MNI (Montreal Neurological Institute) space. Anatomical regions emerging from the between-group comparisons were labelled according to the Anatomy toolbox vers. 2.2c^62^.

For component visualization the source matrix was reshaped back to a 3D-image, scaled to unit standard deviations (Z) and thresholded at Z > 3.0. Maps from the four components described in section 3.2 (see below) were overlaid onto a Montreal Neurological Institute (MNI) normalized anatomical template. Anatomical denominations and stereotaxic coordinates were derived from clusters above a threshold of Z = 3.0 by linking the ICA output images (i.e. the chosen components of interest) to the Talairach Daemon data base (http://www.talairach.org/daemon.html).

#### Functional network connectivity (FNC) analysis

We assessed temporal correlations between RSN (“functional network connectivity”) by means of a constrained maximal lag correlation approach (“Functional Network Connectivity Toolbox”, FNCTB Vers. 2.3, http://mialab.mrn.org/software/fnc/)^31^. Analyses were performed using the prefrontal networks (left and right frontoparietal RSN) and the two DMN components (anterior and posterior DMN), as described below in more detail. To assess functional connectivity between the networks, the individual time courses of the RSN were first interpolated to 50 ms bins to allow detection of sub-TR hemodynamic differences. Subsequently, the temporal correlation for the RSN was estimated by shifting the lag between the time courses from −4.42 to 4.42 seconds (2 TR) and calculating the maximal correlation value and the corresponding lag-value for each participant. After Z-transformation between-group differences of significant maximal correlations and lag-values were compared using two sample t-tests (p < 0.05, uncorrected). A significant correlation between the RSN indicates their temporal dependency whereas the lag values represent the delay between two correlated RSN averaged across each group^31, 63^.

### Correlations between functional connectivity and clinical measures

Correlation analyses within the patient group were calculated between indices of RSN connectivity and clinical measures (p < 0.05, uncorrected for multiple comparisons). Extraction of beta parameters was performed using the MarsBar toolbox (http://marsbar.sourceforge.net) and then processed off-line using the Statistical Package of the Social Sciences (IBM SPSS version 23.0).

### Data availability

All data generated or analysed during this study are included in this published article and its Supplementary Information files.

## Electronic supplementary material

Supplementary Figures

Dataset 1

## References

[1] Rampton D (2009). Does stress influence inflammatory bowel disease? The clinical data. Dig. Dis..

[2] Fuller-Thomson E, Lateef R, Sulman J (2015). Robust Association Between Inflammatory Bowel Disease and Generalized Anxiety Disorder: Findings from a Nationally Representative Canadian Study. Inflamm. Bowel Dis..

[3] Nahon S (2012). Risk factors of anxiety and depression in inflammatory bowel disease. Inflamm. Bowel Dis..

[4] Fuller-Thomson E, Sulman J (2006). Depression and inflammatory bowel disease: findings from two nationally representative Canadian surveys. Inflamm. Bowel Dis..

[5] Dancey CP, Attree EA, Stuart G, Wilson C (2009). & Sonnet, A. Words fail me: the verbal IQ deficit in inflammatory bowel disease and irritable bowel syndrome. Inflamm. Bowel Dis..

[6] Castaneda AE, Tuulio-Henriksson A, Aronen ET, Marttunen M, Kolho KL (2013). Cognitive functioning and depressive symptoms in adolescents with inflammatory bowel disease. World J. Gastroenterol..

[7] Mikocka-Walus A, Andrews JM, Bampton P (2016). Cognitive Behavioral Therapy for IBD. Inflamm. Bowel Dis..

[8] Bonaz BL, Bernstein CN (2013). Brain-gut interactions in inflammatory bowel disease. Gastroenterology.

[9] Thomann AK (2016). Altered Markers of Brain Development in Crohn’s Disease with Extraintestinal Manifestations - A Pilot Study. PLoS One.

[10] Agostini A (2013). New insights into the brain involvement in patients with Crohn’s disease: a voxel-based morphometry study. Neurogastroenterol. Motil..

[11] Bao CH (2015). Alterations in brain grey matter structures in patients with crohn’s disease and their correlation with psychological distress. J Crohns Colitis.

[12] Rubio, A. *et al*. Brain responses to uncertainty about upcoming rectal discomfort in quiescent Crohn’s disease - a fMRI study. *Neurogastroenterol. Motil*., doi:10.1111/nmo.12844 (2016). 10.1111/nmo.12844 27132547

[13] Agostini A (2011). Brain functional changes in patients with ulcerative colitis: a functional magnetic resonance imaging study on emotional processing. Inflamm. Bowel Dis..

[14] Agostini, A. *et al*. Stress and brain functional changes in patients with Crohn’s disease: A functional magnetic resonance imaging study. *Neurogastroenterol. Motil*., doi:10.1111/nmo.13108 (2017). 10.1111/nmo.13108 28560758

[15] Qi, R. *et al*. Intrinsic brain abnormalities in irritable bowel syndrome and effect of anxiety and depression. *Brain Imaging Behav*, doi:10.1007/s11682-015-9478–1 (2015). 10.1007/s11682-015-9478-1 26556814

[16] van den Heuvel MP, Hulshoff Pol HE (2010). Exploring the brain network: a review on resting-state fMRI functional connectivity. Eur. Neuropsychopharmacol..

[17] Buckner RL, Andrews-Hanna JR, Schacter DL (2008). The brain’s default network: anatomy, function, and relevance to disease. Ann. N. Y. Acad. Sci..

[18] Smith SM (2009). Correspondence of the brain’s functional architecture during activation and rest. Proc. Natl. Acad. Sci. USA..

[19] Damoiseaux JS (2006). Consistent resting-state networks across healthy subjects. Proc. Natl. Acad. Sci. USA..

[20] Laird AR (2011). Behavioral interpretations of intrinsic connectivity networks. J. Cogn. Neurosci..

[21] Zhang J (2011). Disrupted brain connectivity networks in drug-naive, first-episode major depressive disorder. Biol. Psychiatry.

[22] Coutinho JF (2016). Default mode network dissociation in depressive and anxiety states. Brain Imaging Behav.

[23] Simon R, Engstrom M (2015). The default mode network as a biomarker for monitoring the therapeutic effects of meditation. Front. Psychol..

[24] Sheline YI, Raichle ME (2013). Resting state functional connectivity in preclinical Alzheimer’s disease. Biol. Psychiatry.

[25] Sambataro F (2010). Treatment with olanzapine is associated with modulation of the default mode network in patients with Schizophrenia. Neuropsychopharmacology.

[26] Greicius MD (2007). Resting-state functional connectivity in major depression: abnormally increased contributions from subgenual cingulate cortex and thalamus. Biol. Psychiatry.

[27] Wang L (2015). The effects of antidepressant treatment on resting-state functional brain networks in patients with major depressive disorder. Hum. Brain Mapp..

[28] Sambataro F, Wolf ND, Pennuto M, Vasic N, Wolf RC (2014). Revisiting default mode network function in major depression: evidence for disrupted subsystem connectivity. Psychol. Med..

[29] Damoiseaux JS, Prater KE, Miller BL, Greicius MD (2012). Functional connectivity tracks clinical deterioration in Alzheimer’s disease. Neurobiol. Aging.

[30] Berrill JW (2013). An observational study of cognitive function in patients with irritable bowel syndrome and inflammatory bowel disease. Neurogastroenterol. Motil..

[31] Jafri MJ, Pearlson GD, Stevens M, Calhoun VD (2008). A method for functional network connectivity among spatially independent resting-state components in schizophrenia. Neuroimage.

[32] Agostini A (2013). Functional magnetic resonance imaging study reveals differences in the habituation to psychological stress in patients with Crohn’s disease versus healthy controls. J. Behav. Med..

[33] Etkin A, Wager TD (2007). Functional neuroimaging of anxiety: a meta-analysis of emotional processing in PTSD, social anxiety disorder, and specific phobia. Am. J. Psychiatry.

[34] Vogt BA (2016). Midcingulate cortex: Structure, connections, homologies, functions and diseases. J. Chem. Neuroanat..

[35] Hamilton JP (2012). Functional neuroimaging of major depressive disorder: a meta-analysis and new integration of base line activation and neural response data. Am. J. Psychiatry.

[36] Yang J (2016). Macro and micro structures in the dorsal anterior cingulate cortex contribute to individual differences in self-monitoring. Brain Imaging Behav.

[37] Herwig U, Kaffenberger T, Schell C, Jancke L, Bruhl AB (2012). Neural activity associated with self-reflection. BMC Neurosci..

[38] Andrews-Hanna JR, Saxe R, Yarkoni T (2014). Contributions of episodic retrieval and mentalizing to autobiographical thought: evidence from functional neuroimaging, resting-state connectivity, and fMRI meta-analyses. Neuroimage.

[39] Xie X (2016). How do you make me feel better? Social cognitive emotion regulation and the default mode network. Neuroimage.

[40] Vogt BA (2005). Pain and emotion interactions in subregions of the cingulate gyrus. Nat. Rev. Neurosci..

[41] Farmer MA, Baliki MN, Apkarian AV (2012). A dynamic network perspective of chronic pain. Neurosci. Lett..

[42] Napadow V (2010). Intrinsic brain connectivity in fibromyalgia is associated with chronic pain intensity. Arthritis Rheum..

[43] Qi, R. *et al*. Topological Reorganization of the Default Mode Network in Irritable Bowel Syndrome. *Mol. Neurobiol*., doi:10.1007/s12035-015-9558-7 (2015). 10.1007/s12035-015-9558-7 26635086

[44] Elsenbruch S (2010). Affective disturbances modulate the neural processing of visceral pain stimuli in irritable bowel syndrome: an fMRI study. Gut.

[45] Baliki MN, Geha PY, Apkarian AV, Chialvo DR (2008). Beyond feeling: chronic pain hurts the brain, disrupting the default-mode network dynamics. J. Neurosci..

[46] Cauda F (2009). Altered resting state in diabetic neuropathic pain. PLoS One.

[47] Hemington, K. S., Wu, Q., Kucyi, A., Inman, R. D. & Davis, K. D. Abnormal cross-network functional connectivity in chronic pain and its association with clinical symptoms. *Brain Struct Funct*, doi:10.1007/s00429-015-1161-1 (2015). 10.1007/s00429-015-1161-1 26669874

[48] Fallon N, Chiu Y, Nurmikko T, Stancak A (2016). Functional Connectivity with the Default Mode Network Is Altered in Fibromyalgia Patients. PLoS One.

[49] Lanting C, Wozniak A, van Dijk P, Langers DR (2016). Tinnitus- and Task-Related Differences in Resting-State Networks. Adv. Exp. Med. Biol..

[50] Bush GC (2011). frontal, and parietal cortical dysfunction in attention-deficit/hyperactivity disorder. Biol. Psychiatry.

[51] Stjernman H, Tysk C, Almer S, Strom M, Hjortswang H (2010). Worries and concerns in a large unselected cohort of patients with Crohn’s disease. Scand. J. Gastroenterol..

[52] Peterson A, Thome J, Frewen P, Lanius RA (2014). Resting-state neuroimaging studies: a new way of identifying differences and similarities among the anxiety disorders?. Can. J. Psychiatry..

[53] Beck, A. T., Steer, R. A. & Brown, G. K. Manual for the Beck Depression Inventory-2. San Antonio, TX: Psychological Corporation. (1996).

[54] Zigmond AS, Snaith RP (1983). The hospital anxiety and depression scale. Acta Psychiatr. Scand..

[55] Flachenecker, P. *et al*. [“Fatigue” in multiple sclerosis. Development and and validation of the “Wurzburger Fatigue Inventory for MS”]. *Nervenarzt* **77**, 165-166, 168–170, 172–164, doi:10.1007/s00115-005-1990-x (2006). 10.1007/s00115-005-1990-x 16160812

[56] Nasreddine ZS (2005). The Montreal Cognitive Assessment, MoCA: a brief screening tool for mild cognitive impairment. J. Am. Geriatr. Soc..

[57] Himberg J, Hyvarinen A, Esposito F (2004). Validating the independent components of neuroimaging time series via clustering and visualization. Neuroimage.

[58] Li YO, Adali T, Calhoun VD (2007). Estimating the number of independent components for functional magnetic resonance imaging data. Hum. Brain Mapp..

[59] Tzourio-Mazoyer N (2002). Automated anatomical labeling of activations in SPM using a macroscopic anatomical parcellation of the MNI MRI single-subject brain. Neuroimage.

[60] Power JD, Barnes KA, Snyder AZ, Schlaggar BL, Petersen SE (2012). Spurious but systematic correlations in functional connectivity MRI networks arise from subject motion. Neuroimage.

[61] Worsley KJ, Cao J, Paus T, Petrides M, Evans AC (1998). Applications of random field theory to functional connectivity. Hum. Brain Mapp..

[62] Eickhoff SB (2007). Assignment of functional activations to probabilistic cytoarchitectonic areas revisited. Neuroimage.

[63] Wolf RC (2009). Temporally anticorrelated brain networks during working memory performance reveal aberrant prefrontal and hippocampal connectivity in patients with schizophrenia. Prog. Neuropsychopharmacol. Biol. Psychiatry.

