# Supplementary material for: Intrinsic neural network dysfunction in quiescent Crohn’s Disease
Source: Sci Rep. 2017 Sep 14;7:11579. doi: 10.1038/s41598-017-11792-y (PMC5599642; doi:10.1038/s41598-017-11792-y)
Supplement: Supplementary file 1 — Supplementary Figures [file 41598_2017_11792_MOESM1_ESM.doc]

**Supplementary figures to the manuscript**

**“Intrinsic neural network dysfunction in quiescent Crohn’s Disease”**

**by Anne K Thomann, Martin Griebe, Philipp A Thomann, Dusan Hirjak, Matthias P Ebert, Kristina Szabo, Wolfgang Reindl and Robert C Wolf**


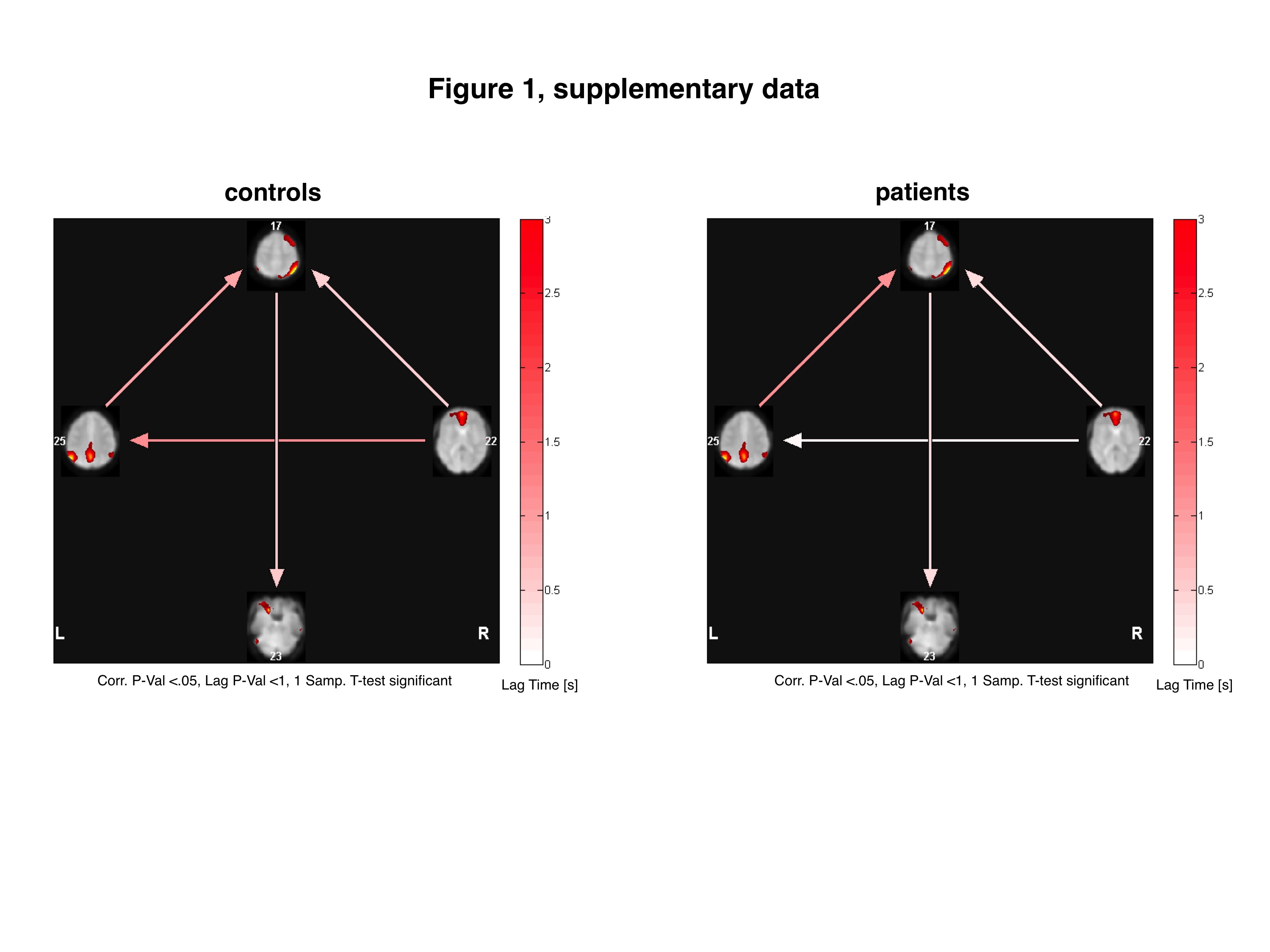


**Figure 1, supplementary data**: Functional network connectivity (FNC) within groups.

The spatial maps and their corresponding arrows indicate significance and directionality of maximal correlations using within- and between-group t-tests (p<0.05, uncorrected). The numbers associated with their corresponding image refer to the respective IC numbers, as obtained by the ICA. Arrow colors reflect the lag time, as indicated by corresponding bars. The difference between the groups was not significant.


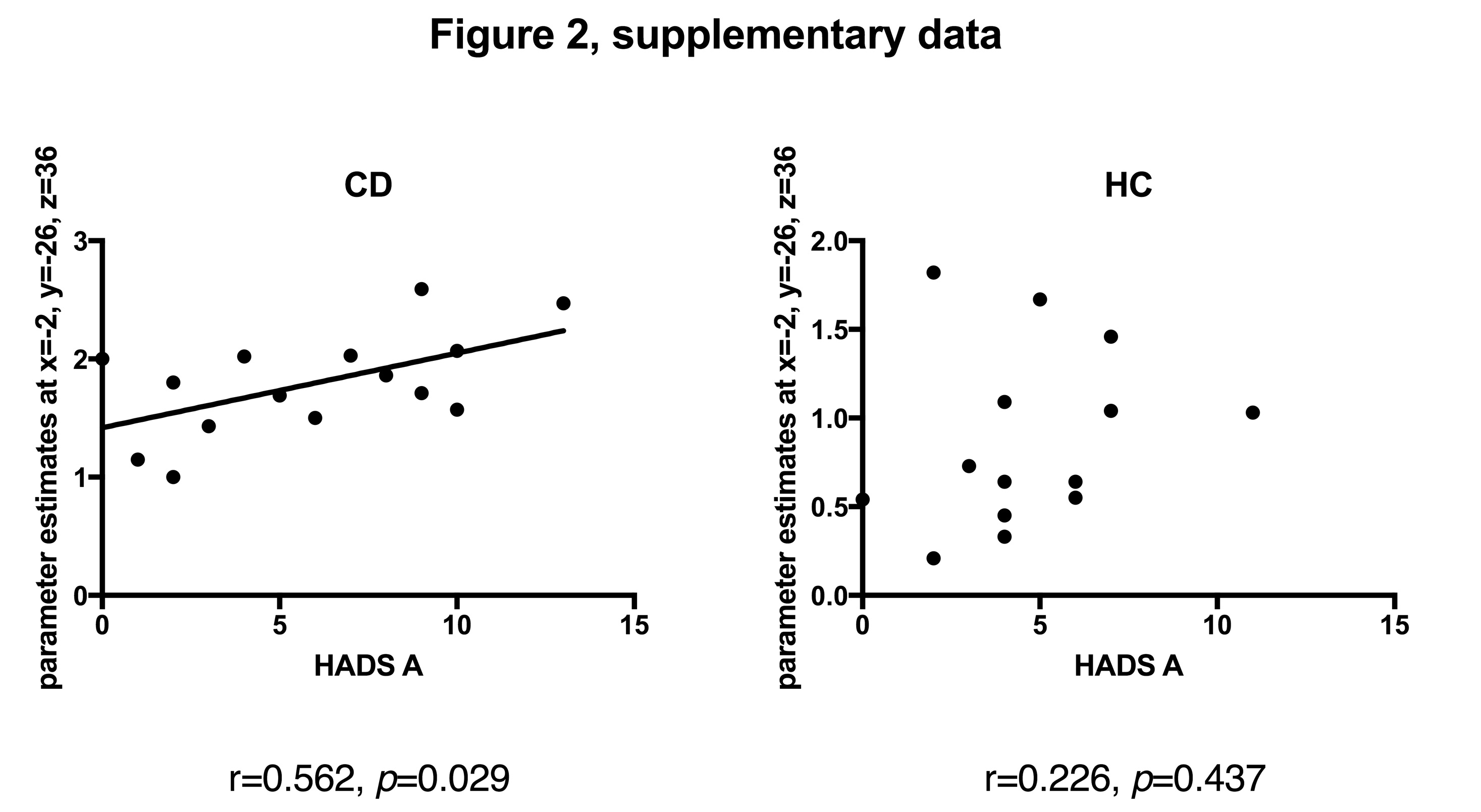


**Figure 2, supplementary data**: Plots showing the relationship between middle cingulate cortex (MCC) connectivity and HADS anxiety scores in patients (CD, r=0.562, p=0.029) and controls (HC, r=0.226, p=0.437).
